# Integrated Membrane Process for the Treatment and Reuse of Residual Table Olive Fermentation Brine and Anaerobically Digested Sludge Centrate

**DOI:** 10.3390/membranes10100253

**Published:** 2020-09-24

**Authors:** Carlos Carbonell-Alcaina, Jose Luis Soler-Cabezas, Amparo Bes-Piá, María Cinta Vincent-Vela, Jose Antonio Mendoza-Roca, Laura Pastor-Alcañiz, Silvia Álvarez-Blanco

**Affiliations:** 1 Instituto de Seguridad Industrial, Radiofísica y Medio Ambiental, Universitat Politècnica de València, Camino de Vera s/n, 46022 Valencia, Spain; carcaral@upvnet.upv.es (C.C.-A.); jsoca@isirym.upv.es (J.L.S.-C.); mbespia@iqn.upv.es (A.B.-P.); mavinve@iqn.upv.es (M.C.V.-V.); jamendoz@iqn.upv.es (J.A.M.-R.); 2 Depuración de Aguas del Mediterráneo (DAM), Avenida Benjamín Franklin 21, Parque Tecnológico, 46980 Paterna, Spain; laura.pastor@dam-aguas.es

**Keywords:** wastewater reuse, table olive fermentation brine, digester centrate, forward osmosis, nanofiltration, ultrafiltration

## Abstract

Management of wastewater is a major challenge nowadays, due to increasing water demand, growing population and more stringent regulations on water quality. Wastewaters from food conservation are especially difficult to treat, since they have high salinity and high organic matter concentration. The aim of this work is the treatment of the effluent from a table olive fermentation process (FTOP) with the aim of reusing it once the organic matter is separated. The process proposed in this work consists of the following membrane-based technologies: Ultrafiltration (UF) (UP005, Microdyn Nadir), Forward Osmosis (FO) (Osmen2521, Hydration Technology Innovation) and Nanofiltration (NF) (NF245, Dow). The FO process was implemented to reduce the salinity entering the NF process, using the FTOP as draw solution and, at the same time, to concentrate the centrate produced in the sludge treatment of a municipal wastewater treatment plant with the aim of obtaining a stream enriched in nutrients. The UF step achieved the elimination of 50% of the chemical oxygen demand of the FTOP. The UF permeate was pumped to the FO system reducing the volume of the anaerobically digested sludge centrate (ADSC) by a factor of 3 in 6.5 h. Finally, the ultrafiltrated FTOP diluted by FO was subjected to NF. The transmembrane pressure needed in the NF stage was 40% lower than that required if the ultrafiltration permeate was directly nanofiltered. By means of the integrated process, the concentration of organic matter and phenolic compounds in the FTOP decreased by 97%. Therefore, the proposed process was able to obtain a treated brine that could be reused in other processes and simultaneously to concentrate a stream, such as the ADSC.

## 1. Introduction

Management of hypersaline wastewaters is very difficult to perform since the high salinity is combined with a high concentration of organic matter. An aqueous effluent is considered hypersaline when its salt concentration is higher than 1% [1]. A source of hypersaline streams can be the production of pickled fruits and vegetables. One of these generated effluents is the fermentation brine from table olive processing (FTOP), whose conductivity can reach 80 ms/cm and its chemical oxygen demand (COD) could be more than 15,000 mg/L [2].

For the brine reuse, the organic matter of the effluent has to be eliminated. The biological treatment is very complicated since salinity reduces the cellular activity significantly [3]. In the case of FTOP, the biodegradation of the organic matter is even slower due to the presence of phenolic compounds (around 1000 mg tyrosol eq·L^−1^) [2]. In this way, the start-up and operation of biological reactors treating this brine is very complicated [4,5].

One alternative for organic matter elimination is advanced oxidation. As complete mineralization is not economically feasible, the combination of advanced oxidation processes with biological treatment could be a better solution. In this way, Ayed et al. [6] reviewed the viability of applying this process combination to FTOP. The disadvantage of this process is the elimination of the phenolic compounds together with the other organic compounds (oil and greases, sugars…). In this way, a further recovery of the phenolic compounds would not be possible.

In contrast to the above-mentioned processes, membrane processes do not oxidize organic matter but may separate it from the brine. Thus, in addition to the brine reuse, the valuable phenolic compounds could be recovered from the FTOP. However, additional processes like adsorption would be necessary [7]. The treatment of FTOP by membrane processes has been scarcely reported in the literature despite the potential interest, which demonstrates the novelty of this work. The works collected from the literature have only tested the membrane process of ultrafiltration [8,9] and membrane distillation [10]. By means of ultrafiltation, the rejection of the COD ranged between 15% and 50% depending on the membranes and operating conditions considered. Thus, the authors indicated that a further treatment by means of other membrane processes, such as nanofiltration or reverse osmosis, or other separation techniques was required. By means of membrane distillation, the FTOP was concentrated and high-quality water was obtained.

In this work, an integrated membrane process has been proposed for the treatment of the FTOP with the aim of reusing the brine and taking advantage of its high osmotic pressure to concentrate a particular stream, as it is the anaerobically digested sludge centrate (ADSC). Three steps were proposed: ultrafiltration, forward osmosis and nanofiltration. Ultrafiltration (UF) and nanofiltration (NF) eliminate the different fractions of organic matter from the FTOP. The use of the FTOP as draw solution in a forward osmosis (FO) process within the integrated process will reduce its conductivity. Therefore, the subsequent NF or reverse osmosis processes would have greater efficiency.

FO is a membrane process based on a natural phenomenon that occurs when a membrane separates two liquids that differ in osmotic pressure. In that case, the solvent (mainly water) crosses the membrane, spontaneously, from the liquid with lower osmotic pressure (named feed solution) to the liquid with higher osmotic pressure (named draw solution, DS). Thus, the concentration of the feed solution and the dilution of the draw solution occur simultaneously. The FO process proposed in this work has the aim of concentrating the anaerobically digested sludge centrate (ADSC), which is an effluent from the sludge dewatering in wastewater treatment plants, which is rich in nutrients. Usually, ADSC is returned to the pretreatment process at the plant entrance, contributing to an increase in organic matter and nutrient concentrations in the wastewater to be treated [11]. The use of wastewaters as DS leads to important cost savings in the FO process, since the DS does not need to be regenerated. In this way, the use of ammonia absorption effluent [12], brines from seawater desalination plants [12], effluents from paper industry containing sodium lignine sulfonate [13] and biogas slurry [14] as DS has been reported by different authors. However, the utilization of FTOP as DS has not been considered in the literature.

In this way, the objective of this work is to assess an integrated process for FTOP management consisting of UF, FO and NF in view of reusing it. At the same time, the FO process will serve to concentrate the ADSC stream, which could be used for agricultural purposes due to the high nitrogen concentration. Until now, these effluents have not been properly treated, being accumulated in evaporation ponds or mixed with other wastewaters for their dilution. Thus, the proposed process could be an appropriate solution for the FTOP management.

## 2. Materials and Methods

### 2.1. Wastewater

As feed solution for the FO step, digester centrate from a municipal wastewater treatment plant placed in Comunidad Valenciana (Spain) was used after filtration at 500 microns.

On the other hand, the residual fermentation brine from table olive production was supplied by a table olive packaging plant in Comunidad Valenciana (Spain). The samples were filtered at 5 µm and they were stored at 5 °C until they were used.

### 2.2. Analytical Methods

A pH-meter (model GLP+21 from Crison, Barcelona, Spain) and an electrical conductivity-meter (model GLP31+ from Crison, Barcelona, Spain) were used to measure the pH and conductivity of the fermentation brine samples treated by membrane processes. Also, two conductivity-meters, model CDH-SD1 from Omega Engineering (Norwalk, CT, USA), were used to measure and register feed and draw solution conductivities.

In order to measure the soluble COD, the samples were previously filtered through a 0.45 µm polytetrafluoroethylene filter. The COD of the filtered samples was determined by means of LCK 114 and LCK 414 kits (Hach Lange, Düsseldorf, Germany). The total nitrogen and the total phosphorous content of the samples were determined with LCK338 and LCK348 kits (Hach Lange, Düsseldorf, Germany), respectively. The total phenolic compounds concentration (expressed in milligrams of tyrosol equivalents per liter; mg tyrosol eq·L^−1^) was determined by means of the Folin–Ciocalteu method [15]. The color of the samples was calculated as the difference between the sample absorbance at 440 and 700 nm according to De Castro and Brenes [16]. The absorbance was measured by means of a DR600 spectrophotometer (Hach Lange, Düsseldorf, Germany).

### 2.3. Integrated Process

**Figure 1:**
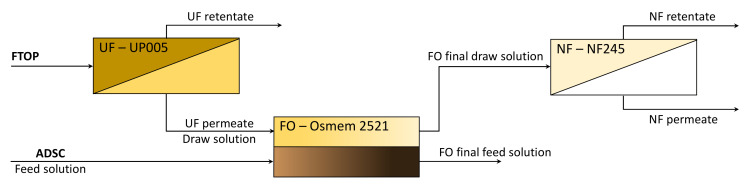
Schematic diagram of the integrated membrane process (FTOP: residual fermentation brine from table olive production; ADSC: anaerobically digested sludge centrate).

A diagram of the integrated membrane process is shown in Figure 1. As can be observed, the wastewater from table olive production was treated by means of an ultrafiltration (UF) process, with the objective of eliminating suspended solids and large molecules and reducing the fouling of the subsequent processes. The UF permeate stream from this process was used as a draw solution in the forward osmosis stage, where the digester centrate was used as the feed solution. In this stage, the objective is to use the osmotic pressure difference between the brine (UF permeate) and the digester centrate to concentrate the last one and to reduce the conductivity of the NF feed. Finally, the diluted brine obtained from the forward osmosis stage was treated by means of a nanofiltration process in order to produce a stream with a lower amount of organic matter. In the following sections, these stages will be described in more detail.

The results obtained in the NF step were compared with those obtained when the UF permeate was submitted to a NF process without the intermediate forward osmosis stage.

All the membrane tests were performed in duplicate, with the largest discrepancy being observed between the permeate fluxes measured lower than 9%. The analytical characterizations were carried out in triplicate.

### 2.4. Ultrafiltration Step

**Figure 2:**
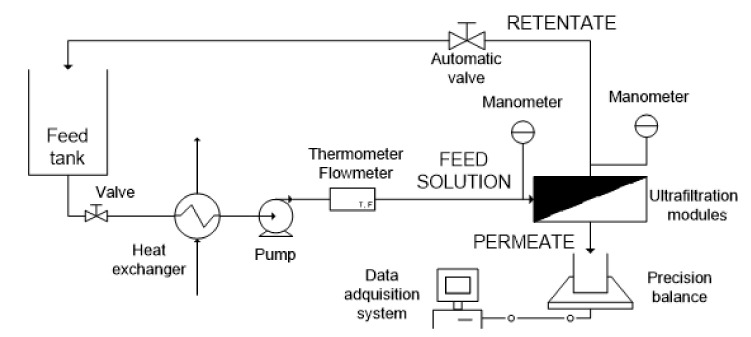
Schematic diagram of the ultrafiltration laboratory plant.

The ultrafiltration unit consisted of an automatic plant where the temperature, the transmembrane pressure (TMP) and the feed flow rate were automatically regulated. Figure 2 shows a diagram of the ultrafiltration laboratory plant. It consists of two Rayflow modules (Orelis Environnement, Salindres, France) connected in series, each one equipped with two UP005 flat sheet membranes (Microdyn Nadir, Wiesbaden, Germany). The total active surface of each flat sheet membrane was 0.0125 m^2^. Therefore, the total active surface was 0.025 m^2^. The feed tank was filled with 20 L of filtered FTOP. The operating conditions were a TMP of 3 bars, a cross-flow velocity (CFV) of 2.2 m·s^−1^ and a temperature of 25 °C. These operating conditions and the membrane were selected according to a previous work [9]. The UF test was carried out in concentration mode (only the retentate was recycled back to the feed tank) up to a volume reduction factor (VRF) of 2.5. The duration of the test was 14.6 h, including a cleaning step in-between. The collected UF permeate was stored at 5 °C prior to being used in the forward osmosis stage. The total permeate flux was calculated from the mass data gravimetrically measured with a Kern PKP precision scale (Kern & Sohn, Balingen, Germany).

The membrane was compacted with osmotic water at 3 bar and the hydraulic permeability was determined at the following operating conditions: TMPs of 1.3, 2.0 and 3.0 bars, cross-flow velocity (CFV) of 2.2 m·s^−1^ and at room temperature.

The apparent solute rejection (*R*) at a certain operating time is usually determined by means of the following equation:

(1) $$R=1-\frac{C_{p}}{C_{F}}$$

where *C_p_* and *C_F_* are the concentration in the permeate stream and in the feed tank at a given operating time.

For batch membrane processes, the concentration of solutes in the retentate stream (*C_R_*) is related to the VRF and solute rejection by means of the following equation [17]:

*C*_*R*_ = *C*_0_ (*VRF*)^*R*^ (2)

where *C_0_* is solute concentration in the initial feed. From this equation, the overall solute rejection was calculated according to:

(3) $$R=\frac{log\left(\frac{C_{R}}{C_{0}}\right)}{log\left(VRF\right)}$$

The cleaning protocol consisted of the following sequence: rinsing with osmotic water, chemical cleaning with NaOH solution at pH 11, rinsing with osmotic water, chemical cleaning with citric acid solution (1% *w*/*v*) and a final rinsing with osmotic water. The operating conditions were TMP of 1.3 bar, CFV of 2.2 m·s^−1^ and at room temperature. The duration of the osmotic water rinsing steps was 9 min without recycling back the permeate and the retentate to the feed tank, whereas the duration of the chemical cleaning steps was 5 min in total recirculation mode (the retentate and permeate were recycled to the feed tank). The chemical cleaning solutions were prepared from pure chemical compounds (Panreac, Barcelona, Spain).

### 2.5. Forward Osmosis Step

**Figure 3:**
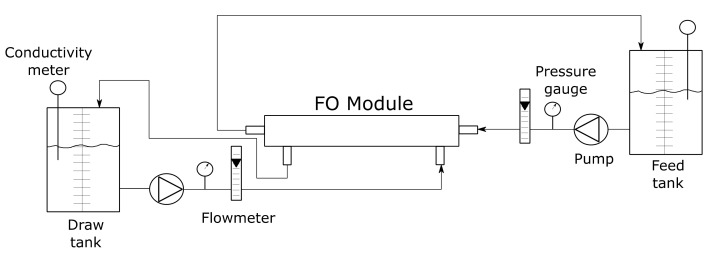
Schematic diagram of the forward osmosis (FO) laboratory plant.

Figure 3 shows a diagram of the forward osmosis laboratory plant.

The membrane used in the FO tests was a spiral wound Osmem 2521FO-CS-CTA-P-3H model from Hydration Technology Innovation (HTI, Albany, NY, USA) with 0.5 m^2^ of active area. A membrane housing, model AXEON 2521 from AXEON Water Technologies (Temecula, CA, USA), was used. A centrifugal pump (model ESPA Tecno 05 from ESPA Innovative Solutions, Spain) was employed to impel the feed solution, whereas a peristaltic pump was used in the case of the draw solution (model Masterflex I/P from Cole-Parmer Instrument Company, Vernon Hills, IL, USA, with a head Easy-Load model 77601-00). The plant contains two flowmeters in order to measure the flow rate of both feed and draw solutions, and two pressure gauges to measure the pressure at the feed and draw solution sides of the membrane. Two 20 L graduated tanks were used to check the volume variations of the draw and feed solutions, respectively.

The FO test started with 5 L in the tank of the draw solution and 15 L in the tank of the feed solution. The flow rates were 42 and 250 L h^−1^ for the draw and feed solutions, respectively. The initial pressures were 0.2 and 0.38 bar for the draw and feed solutions, respectively. The conductivity of draw and feed solutions were registered over time. The test lasted 6 and a half hours and a concentration factor of 3 was reached at the end of the run. Samples of both draw and feed solutions were taken at the beginning and at the end of the test for their characterization.

The experimental water flux (J_w_) and the experimental reverse salt flux (Js) were calculated as described in Reference [18]. The experimental water flux (J_w_) is defined as:

J_w_ = ΔV/(A Δt) (4)

where ‘V’ is the draw solution volume in its tank, ‘A’ is the active membrane area and ‘t’ is the time.

The experimental reverse salt flux (Js) is defined as:

Js = Δm_feed_/(A Δt) = (V_t_ C_t_ − V_0_ C_0_)_feed_/(A Δt) (5)

where ‘m’ is the mass of salts in the feed solution tank, ‘V’ is the feed solution volume in the tank and ‘C’ is the salt concentration in the feed solution tank.

### 2.6. Nanofiltration Step

**Figure 4:**
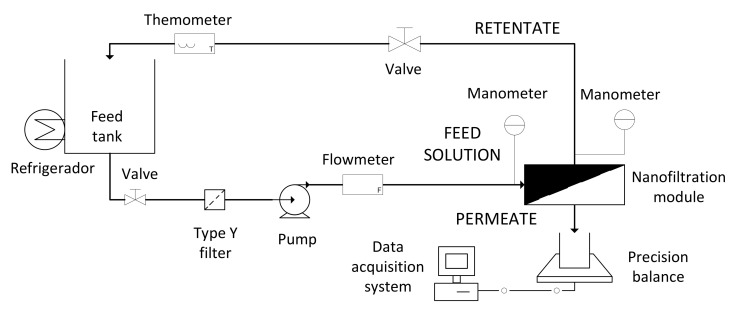
Schematic diagram of the nanofiltration (NF) laboratory plant.

The NF tests were carried out in the NF laboratory plant shown in Figure 4. It was semiautomatic, with CFV and temperature being automatically controlled.

The membrane module was equipped with a NF245 (Dow Chemical, Midland, MI, USA) polyamide flat sheet membrane, with an active surface of 0.0047 m^2^. The membrane and operating conditions were selected after a previous work performed by the authors and taking into account the recommendations by the manufacturer [19]. The module was designed in the Research Institute for Industrial, Radiophysical and Environmental Safety (ISIRYM, Valencia, Spain) [20]. The NF membrane was compacted with osmotic water at 20 bar and its initial hydraulic permeability was determined at the TMPs of 5, 10 and 15 bars, a CFV of 2.2 m·s^−1^ and at room temperature. 

The NF tests were performed in total recirculation mode. In this pilot plant, two different streams were tested: the UF permeate and the diluted draw solution from the FO step. The operating conditions in all the NF tests were 5 L of feed volume, CFV of 1.5 m·s^−1^ and temperature of 25 °C. The TMP considered when the UF permeate was treated was 15 bar, while two different TMPs (9 and 15 bar) were tested when the diluted draw solution was treated.

The protocol to clean the NF membrane consisted of sequential rinsing with tap and osmotic water. The operating conditions were no TMP, CFV of 1.0 m·s^−1^ and room temperature. At the beginning, the rinsing was performed in non-recycling mode. The tap water rising step lasted until the conductivity of the retentate stream had the same value as tap water conductivity. The rinsing step carried out with osmotic water was performed until the conductivity of the retentate was the same as that of the osmotic water. Finally, the system was configured in retentate recycling mode and the osmotic water rising was performed until the permeate had the same conductivity as the osmotic water. After each cleaning cycle, the hydraulic permeability of the membrane was checked at a TMP of 10 bar, a CFV of 2.2 m·s^−1^ and room temperature. If the value was greater than 95% of the initial hydraulic permeability, it was considered that the cleaning protocol was effective, otherwise the membrane cleaning was repeated. The NF membranes were cleaned at the end of each run.

## 3. Results and Discussion

### 3.1. Characterization of Feed Samples

The characterization of the feed streams treated in this work (digester centrate and residual FTOP) is presented in Table 1. As it can be observed, the composition of the wastewater from table olive production (UF feed) showed the typical characteristics of this type of wastewater: acidic pH (around 4.0), high conductivity (50 to 95 mS cm^−1^), large amount of dissolved COD (6000 to 35,000 mg O_2_ L^−1^) and high concentration of phenolic compounds (500 to 2000 mg of tyrosol equivalents L^−1^) [9,19,21]. Also, the total nitrogen and phosporous content are consistent to the ones reported in the bibliography (200 to 500 and 23 to 60 mg L^−1^, respectively) [19].

On the other hand, the composition of the ADSC showed a neutral pH (7.8), a low, but not negligible, conductivity (11.9 mS·cm^−1^), a COD of 613 mg·O_2_ L^−1^, a total nitrogen concentration of 1087 mg·L^−1^, a total phosphorous concentration of 9.7 mg·L^−1^ and total phenolic compounds concentration of 63.4 mg of tyrosol equivalents L^−1^. Soler-Cabezas et al. reported the characterization of other ADSC. They found similar values to the ones shown in Table 1, with the only exception of COD, which was higher [12].

### 3.2. Ultrafiltration Step

The hydraulic permeability of the UF membranes determined in this work was 28.4 ± 2.7 L·h^−1^·m^−2^·bar^−1^ at 25 °C, which complies with the specifications given by the manufacturer.

**Figure 5:**
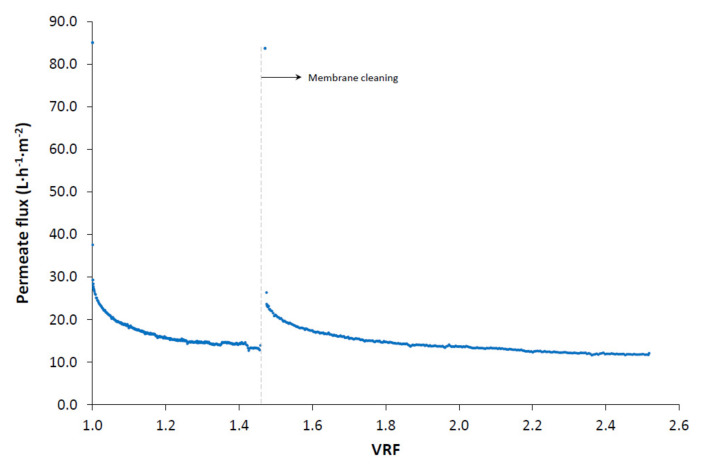
Evolution of permeate flux during the ultrafiltration step as a function of the volume reduction factor.

Figure 5 shows the evolution of permeate flux during the UF step as a function of the VRF. A sharp decline in the permeate flux can be observed at the beginning of the test, which may be indicative of a phenomenon of pore blockage fouling. The subsequent gradual decrease in permeate flux is indicative of an accumulation of particles and molecules on the surface of the membrane, forming a cake and gel layer respectively, as reported by Carbonell-Alcaina et al. [22]. Despite the initial sharp permeate flux decline due to pore blocking and adsorption phenomena, the permeate flux decline is mainly caused by fouling due to cake layer formation, as demonstrated by Carbonell-Alcaina et al. [22]. At the end of the run and when the VRF reached a value of 1.46, the membranes were cleaned. The cleaning protocol proposed recovered 95% of the initial membrane hydraulic permeability.

The characterization of the total amount of permeate collected is shown in Table 1. As it can be noted, the UP005 membrane mainly retained organic matter (COD rejection was equal to 51.9% ± 0.4%), while the rejection of phenolic compounds was 35.9% ± 3.7%. Likewise, the rejection index of nitrogenous compounds was of about 0.4% ± 13.4%. On the other hand, the concentration of phosphorous compounds in the total permeate obtained and in the final retentate were practically the same. However, those concentrations were lower than feed concentration. If a material balance is applied, the amounts of phosphorous compounds before starting the test (in the feed solution) was greater than that at the end of the test (in the permeate and retentate finally obtained). The reason can be the trend of these compounds to precipitate as calcium phosphate as their concentration increases in the vicinity of the membrane surface due to the effect of concentration polarization [12].

### 3.3. Forward Osmosis Step

**Figure 6:**
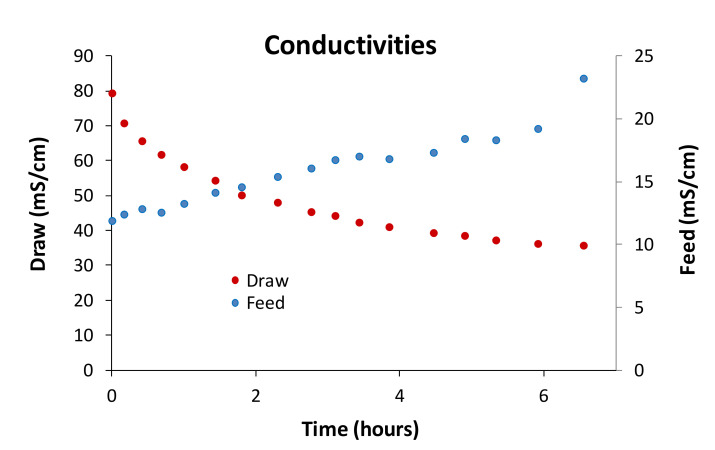
Conductivity of feed and draw solutions versus time in the forward osmosis step.

Figure 6 shows the evolution of the conductivity with the operating time in both the feed solution (ADSC) and the draw solution (UF permeate). It can be observed that the conductivity of the draw solution was reduced by a half in 6.5 h, while the conductivity of the feed solution increased twice the initial value.

**Figure 7:**
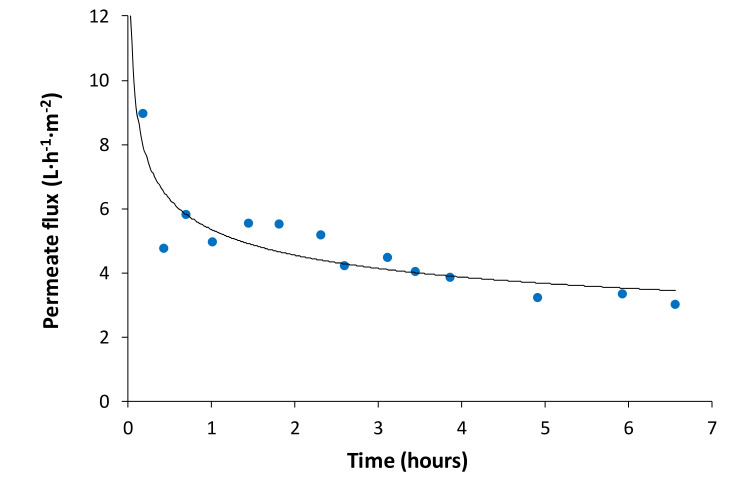
Permeate flux versus time in the forward osmosis step.

Figure 7 shows the evolution of water flux over time. It can be seen that there is a reduction in water flux as expected. This reduction can be attributed to membrane fouling and to the reduction in the driving force (osmotic pressure difference between both sides of the membrane) with time. The initial sharp decay in water flux was due to membrane fouling and not to a dilution effect. This is in accordance with the forward osmosis study performed by Soler-Cabezas et al., who used anaerobically digested sludge centrate as feed and desalination brine (conductivity of 84.7 ms/cm) as draw solution [12] in a FO process. The results showed that water flux diminished from 5.5 to 4 L·m^−2^·h^−1^ in 10 h. The initial flux decay observed by these authors was lower than that observed in this work. This implies that presumably, Figure 7 is showing a fouling effect at the beginning of the test.

Table 1 shows the characterization of both the draw and feed solutions at the beginning and at the end of the forward osmosis test.

Regarding the evolution of pH versus time, feed pH remained practically constant, but the pH of the draw solution exhibited an upward trend (from a pH value of 4.5 to a final value of 8.5). This may be due to volatile acids removal from the fermentation brine, which may be explained by back diffusion to the membrane feed side and to oxidation phenomena. Ferrer-Polonio et al. also found an increase in the pH in a sequencing batch reactor that treated residual brine from table olive production, which was attributed to the biodegradation of the organic acids that are present in the brine [19].

As can be seen from Table 1, during the forward osmosis process, the conductivity and COD met the material balance between the input and output. However, the same did not happen in the case of phenolic compounds, nitrogen and phosphorous. Taking into account that the pH of the draw solution increased to a basic pH, it can be considered that a degradation by oxidation of phenolic compounds could have occurred. This assumption could be confirmed by an increase in the color of the draw solution [19,23].

It is also worth noting that the final phosphorus concentration in the feed solution was lower than that at the beginning. This fact can be explained by phosphorus precipitation in the feed tank, as calcium was observed to cross the membrane from the draw to the feed solution due to reverse salt permeation, thus forming calcium phosphate, as reported by Soler-Cabezas et al. [12]. As previously commented, the material balance was not met in the case of phosphorus, which confirms the phosphorus precipitation.

The feed solution was dehydrated by a factor of three and the draw solution was diluted by a factor of three at the end of the forward osmosis test (after 6.5 h). Concerning nitrogen, although it was not concentrated as expected (three times), a concentration of 1642 mg L^−1^ in the final feed solution was obtained. It means that the rejection of nitrogen was not 100%. This was also observed by Li et al., who used different FO membranes for the concentration of digested manure centrate and reported that ammonium nitrogen was only retained by approximately 40% [24]. Nevertheless, the concentration of nitrogen at the end of the test was greater than that in the ADSC and it was high enough to consider the digested centrate as a source of nitrogen for its further use.

### 3.4. Nanofiltration Step

The initial hydraulic permeability of the NF245 membrane was 6.2 ± 0.3 L·h^−1^·m^−2^·bar^−1^ at 25 °C. This value was slightly lower than that provided by the manufacturer (8.6 L·h^−1^·m^−2^·bar^−1^). This could be due to the small size of the tested membrane area and to the fact that the density of the active layer of NF membranes is not completely homogeneous [25].

**Figure 8:**
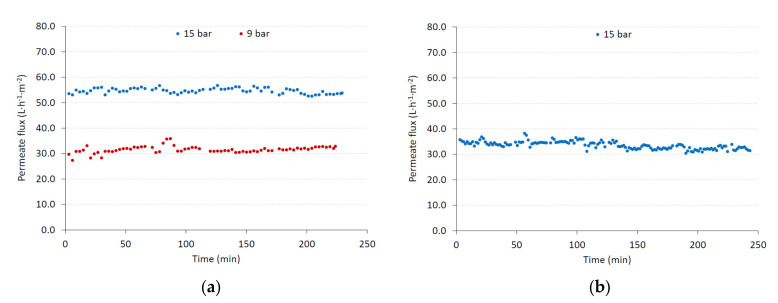
Evolution of permeate flux with time during the nanofiltration step: (**a**) the feed solution was the diluted draw solution from the forward osmosis step, and (**b**) the feed solution was the ultrafiltration permeate.

Figure 8a shows the permeate fluxes obtained at 15 and 9 bar when the diluted draw solution from the FO step was nanofiltered. These results were compared with those obtained at 15 bar when the permeate from the UF step was nanofiltered without an intermediate FO step (Figure 8b).

Firstly, it can be noted that, in all the runs, the evolution of the permeate flux with time did not show the typical decline, although the permeate flux was much lower than pure water permeate flux. Compared with water permeate flux, it decreased by about 65% when the UF permeate was nanofiltered, while it decreased by about 42% when the diluted draw solution was nanofiltered. This can be attributed to the high salt concentration, as also observed by other authors [26]. These authors tested the DS5 DK nanofiltration membrane with solutions that contained dyes with different NaCl concentrations (between 1 to 80 g·L^−1^) and observed a similar trend at salt concentrations higher than 40 g·L^−1^. The conductivity of the FTOP corresponds, approximately, to a NaCl concentration of 50 g·L^−1^. According to these authors [26], a high salt concentration on the membrane surface reduces the membrane fouling due to cake layer formation on its surface. Also, a high concentration of salts on the surface of the membrane increases its hydrophilicity, as observed by a great number of authors, reducing the trend to cake layer formation [27]. Virga et al. studied the effect of ionic strength on the wettability of amphoteric surfaces, as is the case of polyamide membranes [28]. They observed that, as salt concentration increased, the surface became more hydrophilic. They explained this observation by the influence of salt concentration on the Debye length, and thus on the thickness of the electrical double layer (EDL). An increase in the ionic strength causes a reduction in EDL thickness, which turns into a concentration of H^+^ or OH^–^ at the surface that is much closer to the one in the bulk. Thus, if the ionic strength increases, with the pH being constant, the ionization of the surface groups increases too [28]. An increase in the surface charge of the membrane surface translates into greater membrane hydrophilicity [26]. The consideration that membrane fouling is small is supported by the constant value of permeate flux observed during the tests performed in this work. The membrane cleaning process reinforced this hypothesis, since, with a simple water rinsing, the initial hydraulic permeability of the membrane was fully recovered. Therefore, the small permeate flux observed during the NF tests can be mainly attributed to the effect of the osmotic pressure gradient across the membrane caused by the high concentration of salts in the feed [29].

If the steady-state permeate fluxes obtained for the two tests at 15 bar are compared, it can be noted that the flux value is remarkably higher for the diluted draw solution from the FO step. In order to obtain a steady-state permeate flux similar to that obtained when treating the UF permeate, the TMP can be reduced to 9 bar, which considerably decreases the energy costs of the NF step.

In addition, as observed in Table 2, the rejection of the different compounds was significantly higher in the case of the diluted draw solution from the FO stage (dilution factor 1:3). In reverse osmosis and NF processes, rejection usually decreases with concentration due to the increase in the osmotic pressure gradient and the subsequent decrease in the solvent permeate flux, according to the Kedem–Splieger model [30]:

J_w_ = L_p,w_ (ΔP − σ Δπ) (6)

where J_w_ is the permeate flux, L_p,w_ is the water permeability of the membrane, ΔP is the TMP, σ is the reflection coefficient and Δπ is the osmotic pressure gradient across the membrane.

Moreover, rejection was observed to increase with pressure, as was also expected.

Finally, as can be observed from Table 1, the NF permeate obtained is a brine with a low concentration of COD and phenolic compounds, which could favor its treatment in a wastewater treatment plant (WWTP) or be reused for other purposes.

## 4. Conclusions

In the ultrafiltration of the FTOP, the organic matter was partially retained (rejection index of 51.9% and 35.9% for COD and phenolic compounds, respectively). However, the rejection of nitrogen was negligible and phosphorous compounds were suspected to be precipitated as calcium phosphate.

Regarding the forward osmosis step, feed and draw solution volumes were reduced and incremented respectively, by a factor of 3 in 6.5 h. Nitrogen was not completely retained by the membrane, but its concentration at the end of the test was great enough to consider the concentrated ADSC as a source of nitrogen for its further use. In addition, phosphorus compounds were observed to precipitate as well.

In the nanofiltration step, the rejection of COD and phenolic compounds were close to 89% and 85% respectively, while the color of the samples was practically eliminated. Significantly greater fluxes were obtained in comparison with the nanofiltration of the UF permeate without the intermediate FO step.

Finally, the final NF permeate from the integrated process obtained at 9 bar and a cross-flow velocity of 1.5 m s^−1^ showed a reduction in the concentration of COD and phenolic compounds of around 97% when compared with the FTOP. This final brine had low values of COD (331 mg O_2_·L^−1^) and phenolic compounds concentration (25.9 ± 0.5 mg·L^−1^ Ty eq.), which favors its treatment in a WWTP or allows reusing for other purposes.

Experiments at higher scale have to be performed to check the economic feasibility of the integrated process. Fouling of the UF membrane at medium–long-term time and the management of the retentate streams from the UF and NF steps will be key factors to implement this integrated process.

## Figures and Tables

**Table 1:** Characterization of the different streams (FTOP: residual fermentation brine from table olive production).

	Ultrafiltration (UF)					Nanofiltration (NF)		
			Forward Osmosis (FO)					
	UF Feed (FTOP)	UF Retentate	UF Permeate FO Draw Solution	FO Initial Feed (Digester Centrate)	FO Final Feed (Digester Centrate)	FO Final Draw Solution NF Feed	NF Permeate (15 bar)	NF Permeate (9 bar)
pH	4.4 ± 0.2	4.8 ± 0.1	4.5 ± 0.1	7.8 ± 0.1	8.1 ± 0.1	8.8 ± 0.2	8.9 ± 0.1	8.7 ± 0.1
Conductivity (mS cm^−1^)	72.3 ± 0.2	79.3 ± 0.2	71.5 ± 0.9	11.9 ± 0.2	23.2 ± 0.2	25.7 ± 0.2	21.0 ± 0.2	24.5 ± 0.1
Color	0.458 ± 0.002	0.558 ± 0.002	0.252 ± 0.016	-	-	0.838 ± 0.053	0.001 ± 0.000	0.003 ± 0.001
COD ^1^ (mg O_2_ L^−1^)	9730 ± 57	15650 ± 260	7756 ± 67	613 ± 28	1740 ± 103	2663 ± 68	301 ± 4	331 ± 4
Total nitrogen (mg L^−1^)	474.0 ± 28.3	475.8 ± 30.1	421.2 ± 26.5	1087.9 ± 45.4	1642.4 ± 45.4	654.0 ± 31.2	274.0 ± 25.6	317.0 ± 24.7
Total phosporous (mg L^−1^)	37.2 ± 2.1	29.6 ± 1.5	28.9 ± 1.6	9.7 ± 0.5	2.7 ± 0.5	7.0 ± 0.2	N.d ^3^	N.d
Total ph. comp. ^2^ (mg·Ty eq. L^−1^)	1012.6 ± 24.3	1407.3 ± 13.4	812.9 ± 30.2	63.4 ± 1.6	154.7 ± 4.9	152.0 ± 27.7	23.2 ± 2.0	25.9 ± 0.5

^1^ COD: chemical oxygen demand; ^2^ ph. comp.: phenolic compounds; ^3^ N.d.: non detected.

**Table 2:** Solutes rejection in the nanofiltration step (UF-FO-NF: the feed solution was the diluted draw solution from the FO step; UF-NF: the feed solution was the UF permeate).

	UF-FO-NF (15 bar)	UF-FO-NF (9 bar)	UF-NF (15 bar)
Conductivity (%)	18.3 ± 1.2	4.7 ± 1.0	25.4 ± 0.3
Color (%)	99.9 ± 0.1	99.6 ± 0.2	91.4 ± 1.2
COD (%)	88.7 ± 0.4	87.6 ± 0.4	64.7 ± 0.6
Total nitrogen (%)	58.1 ± 5.1	51.5 ± 5.2	-
Total phosporous (%)	100.0 ± 0.0	100.0 ± 0.0	-
Total phenolic compounds (%)	84.7 ± 3.7	83.0 ± 3.8	35.2 ± 2.4
